# Harnessing endophytic fungi for sustainable agriculture: ecological roles, mechanisms, and future prospects

**DOI:** 10.3389/fmicb.2026.1710071

**Published:** 2026-03-09

**Authors:** Changliang Du, Qian Chen, Dayong Cui, Muhammad Zahid Mumtaz, Yonglan Chang, Ning Yang, Liwen Wang, Jie Gao, Weiyi Feng, Junke Zhu

**Affiliations:** 1 Shandong Engineering Research Center of Rose Breeding Technology and Germplasm Innovation, School of Life Sciences, Qilu Normal University, Jinan, China; 2 State Key Laboratory of Aridland Crop Science, College of Agronomy, Gansu Agricultural University, Lanzhou, China; 3 Institute of Molecular Biology and Biotechnology, The University of Lahore, Lahore, Pakistan

**Keywords:** agricultural sustainability, crop growth, endophytic fungi, nutrient uptake, stress tolerance

## Abstract

Endophytic fungi are asymptomatic microorganisms that inhabit plant tissues and play pivotal roles in regulating crop growth under field conditions. This review first provides an overview of their taxonomy and ecological functions, emphasizing natural diversity and distribution, then systematically summarizes their core mechanisms: enhancing nutrient uptake, regulating phytohormone biosynthesis, promoting root development, and boosting resistance to abiotic stresses (e.g., drought, salinity). We further discuss the agricultural potential and existing challenges, including stability, persistence, and compatibility with current farming practices. Future research directions are outlined to advance sustainable agriculture, focusing on dissecting molecular interactions between endophytic fungi and crops, optimizing application techniques, and evaluating long-term ecological impacts. This work provides a comprehensive reference for agricultural scientists, ecologists, and researchers to facilitate the practical application of endophytic fungi, encouraging further research, and practical applications in this field.

## Graphical Abstract

**Figure:**
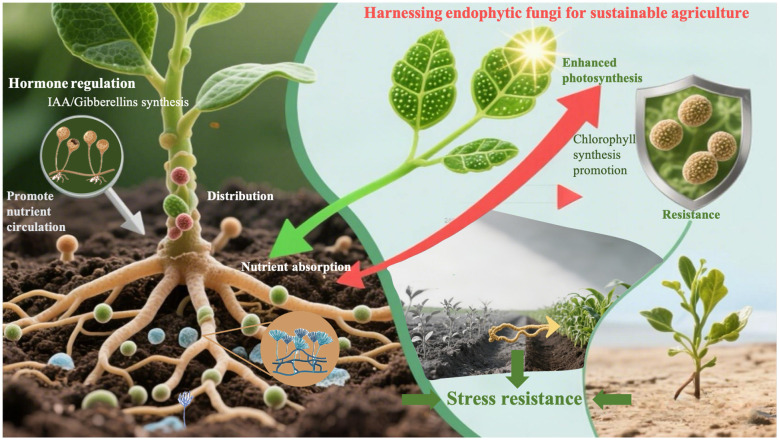

## 1 Introduction

The agricultural sector faces unprecedented challenges due to global population growth and climate change (Rai et al., 2023). To meet the rising food demand while minimizing environmental impacts, researchers are exploring a variety of innovative agricultural technologies. One promising area is endophytic fungi, microorganisms that form symbiotic relationships with plants (Ralph and Hannah, 2015). Endophytic fungi are widely distributed in nature, inhabiting various plants, including crops, herbs, and tree species. Their survival strategies and ecological roles are diverse, encompassing nutrient competition, antibiosis, and phytohormone regulation (Sarkar et al., 2021). These fungi have attracted significant scientific attention due to their potential to promote crop growth and enhance plant stress tolerance (Yan et al., 2019).

Research on endophytic fungi has advanced significantly over the past century: early 20th-century studies focused on basic biology and ecological functions (Qin et al., 2024); by the 1970s−1980s, researchers recognized their potential to promote plant growth and protect against pathogens (Schmidt, 2010); and recent work has shifted to exploring plant-fungus interactions and agricultural applications (Wen et al., 2022; Yan et al., 2019). Since then, advancements in molecular biology and genetics have enabled deeper exploration of their interactions with plants.

Research on endophytic fungi has evolved significantly since their discovery in the early 1900s (White et al., 2019). Early 20th-century studies focused on their basic biology and ecological functions, with growing recognition of their plant growth-promoting and disease-resistant potential in the 1970s to the 1980s (Compant et al., 2016). Advances in molecular biology during the 1990s−2000s enabled precise identification, classification, and revelation of their diversity (Poveda et al., 2022). Since 2010, research has shifted toward agricultural applications, emphasizing the mechanisms underlying growth promotion and stress resistance (Yan et al., 2019).

Endophytic fungi form distinct symbiotic relationships with plants, exerting positive effects on nutrient absorption, hormonal regulation, and root development (Yan et al., 2019; Qin et al., 2024). Some endophytic fungi produce antibiotics to suppress competing microorganisms, thus safeguarding the host plant from pathogens (Rahnama and Ebrahimzadeh, 2006). Certain endophytic fungi synthesize phytohormones like growth promoters and cytokines, contributing to the plant's growth and development (Rigobelo and Baron, 2022). They also enhance crop resilience to biotic and abiotic stresses (Waqas et al., 2013), yet their application in agriculture is hindered by unresolved challenges: unclear molecular mechanisms in field settings, poor stability and compatibility with existing practices, and insufficient understanding of long-term ecological impacts (Nasif et al., 2023). However, the specific mechanisms by which endophytic fungi affect crop growth in real-world agricultural settings are still not fully understood. This knowledge gap limits the effective utilization of their benefits, highlighting the need for systematic exploration.

The review aims to explore the various mechanisms by which endophytic fungi influence crop growth in field conditions. It will cover their classification, ecological functions, relationship with crop growth, role in enhancing crop stress resistance, as well as the potential benefits and challenges of their agricultural application. The review will also propose future research directions to improve our understanding and optimization of endophytic fungi in agriculture. By deepening our knowledge of crop-endophyte interactions, we aim to enhance crop yield and quality and promote sustainable agricultural practices.

## 2 Ecological functions and roles of endophytic fungi

### 2.1 The classification and ecological significance of endophytic fungi

Endophytic fungi reside in various plant structures (roots, stems, leaves) and maintain symbiotic relationships with hosts throughout the plant's life cycle or specific stages. The term “endophytic fungi” was first introduced by Bary in 1866, and later defined as asymptomatic microorganisms that do not induce pathological changes in the host during symbiosis (Aamir et al., 2020). They play crucial roles in enhancing plant growth, improving health, and increasing disease resistance (Khiralla et al., 2016; Wen et al., 2022).

**Figure 1:**
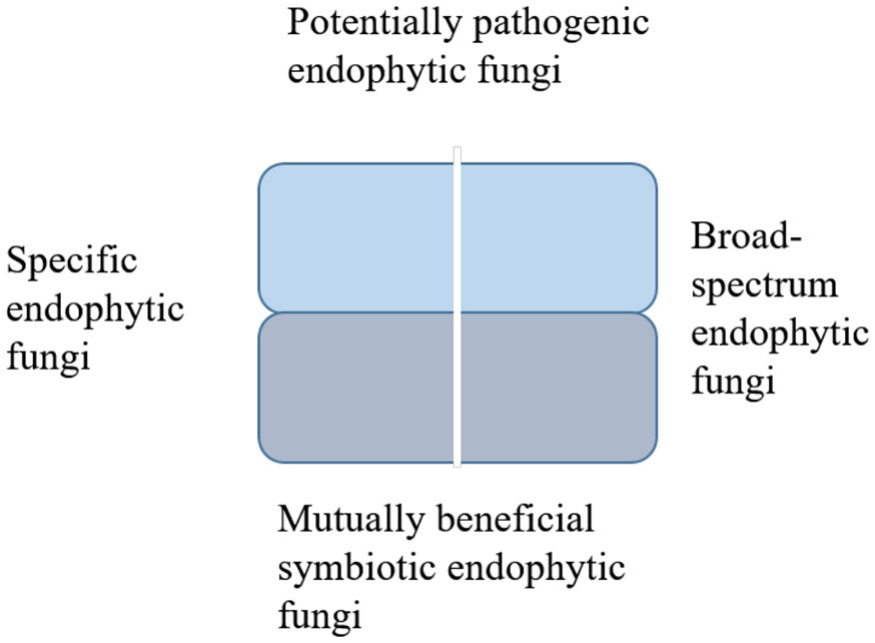
Hierarchical classification of endophytic fungi based on ecological type, distribution, and host specificity.

Endophytic fungi may be classified across multiple dimensions, including ecological function, distribution characteristics, and host specificity (Figure 1). Within the ecological dimension, they may be categorized as mutualistic symbionts or latent pathogens; within the distribution dimension, they may be classified as systemic or localized. Ecologically, they are divided into mutually beneficial symbiotic fungi (aiding nutrient absorption and stress resistance) and potentially pathogenic fungi (harmful under specific conditions). By distribution and function, they are categorized as systemic (persisting throughout the plant's life cycle) or localized (confined to specific plant parts). Host-specificity-based classification distinguishes between specific endophytes (adapted to single plant species) and broad-spectrum endophytes (thriving in multiple hosts).

Rodriguez et al. (2009) proposed a functional classification system dividing endophytic fungi into four classes based on host specificity, transmission mode, and ecological roles (Table 1). Class 1 comprises clavicipitaceous fungi (e.g., *Epichloë spp*.), exclusive to grasses, with vertical and horizontal transmission, producing alkaloids to deter herbivores and pathogens (Hyde and Soytong, 2008). Class 2 endophytes (e.g., *Trichoderma spp*.) colonize aerial tissues and roots of non-grass hosts, promoting growth via phytohormones (IAA, gibberellins) and inducing systemic resistance (Dutta and Banerjee, 2024). Class 3 fungi (e.g., *Colletotrichum spp*.) inhabit the leaves of tropical treesand rely solely on horizontal transmission through rain splash or insect vectors. They enhance photosynthetic efficiency under low-light conditions but do not exhibit systemic colonization (Bamisile et al., 2018; Dutta and Banerjee, 2024). Class 3 fungi (e.g., *Colletotrichum spp*.) inhabit tropical tree leaves, relying on horizontal transmission and enhancing photosynthetic efficiency under low-light conditions (Bamisile et al., 2018). Class 4 comprises dark septate endophytes (DSEs, e.g., *Phialocephala spp*.) in roots, which solubilize phosphorus and improving drought tolerance through osmolyte accumulation (Kamran et al., 2022). Recently, Lugtenberg et al. (2016) added a fifth class for entomopathogenic fungi (e.g., *Beauveria spp*.) that colonize both plants and insects, providing pest control and growth promotion (Verma et al., 2024). These classification systems lay the foundation for understanding the functional mechanisms of endophytic fungi, which are detailed in Section 3.

**Table 1:** Functional classification of endophytic fungi (Rodriguez scheme, 4-class version) and their core ecological services.

**Class**	**Representative genera**	**Parasitic position**	**Transmission**	**References**
1	*Metarhizium, Epichloe, Claciceps*, et al.	Grass, buds and roots	Horizontal & vertical	Behie et al., 2015; Faeth and Saari, 2012; Zhang et al., 2021
2	*Ascomycetes, Penicillium, Aspergillus, Fusarium, Aspergillus, Xylomycetes*, et al.	Plants, roots, stems, leaves, etc.	Horizontal & vertical	Hiruma et al., 2018; Mustafa et al., 2009; Waqas et al., 2013
3	*Ascomycetes, Penicillium, Aspergillus, Fusarium, Aspergillus, Xylomycetes*, et al.	Tropical trees, leaves	Horizontal only	Hiruma et al., 2018
4	*Curvularia, Alternaria, Phialocephala, Deschlera, Ophiosphaerella, Cladosporium*, et al.	Strong host range, roots	Horizontal only	Hiruma et al., 2018; Ikram et al., 2018; Khan et al., 2011; Mehmood et al., 2018

Transcriptomic studies reveal that colonization by endophytic fungi such as *Serendipita indica*, triggers systemic transcriptional reprogramming in host plants. This includes the upregulation of jasmonic acid (JA) and salicylic acid (SA)-responsive genes, such as *PDF1.2* and *PR1*, as well as transporters like *NRT1.1* and *PHT1* (Verma et al., 2024). Additionally, metabolomic profiling demonstrates that these fungal induce the accumulation of stress-protective compounds including proline and flavonoids under drought and salinity stress (Morales-Vargas et al., 2024). These changes are mediated by *MAPK* signaling pathways, specifically *MPK3* and *MPK6*), and reactive oxygen species (ROS)-scavenging enzymes, such as superoxide dismutase (SOD) and catalase (CAT), as confirmed by quantitative PCR (qPCR) and liquid chromatography-tandem mass spectrometry LC-MS/MS analyses (Leetanasaksakul et al., 2024). The transition between mutualistic and latent pathogenic roles of endophytes is not static but mediated by biotic and abiotic factors. For example, *Epichloë spp*. (Class 1 endophytes) produce alkaloids that deter herbivores in healthy grasses but can reduce host fitness under nutrient-poor conditions by competing for carbon resources (Hyde and Soytong, 2008). Similarly, *Phialocephala spp*. (DSEs, Class 4) solubilize phosphorus and improve drought tolerance in wheat (Kamran et al., 2022) but induce root rot in barley when soil moisture exceeds 80% field capacity (Li et al., 2019). Conflicting taxonomic reports further complicate this distinction: some studies classify *Colletotrichum gloeosporioides* as a mutualist that enhances photosynthetic efficiency (Bamisile et al., 2018), while others identify it as a latent pathogen that causes anthracnose in stressed tomato plants (Dutta and Banerjee, 2024). This inconsistency may stem from intraspecific variability in fungal strains, as well as differences in host genotype and environmental conditions.

### 2.2 Functional roles in plant growth and stress tolerance

The mutualistic relationship between endophytic fungi and host plants enhances plant characteristics and ecological success (Venkatesan et al., 2016). These fungi exhibit high ecological diversity, colonizing various plant species and adapting to a wide range of environmental conditions. The diversity and population density of endophytic fungi can vary significantly across different plant taxa; for instance, robust, dense leaves often harbor less fungal diversity and fewer endophytic individuals than delicate, pliable leaves, suggesting that leaf traits may influence fungal distribution (Tellez et al., 2022). Within ecosystems, endophytic fungi occupy specific ecological niches and interact with host plant components such as roots, leaves, and stems, significantly affecting plant growth and vitality. Furthermore, endophytic fungi play a crucial role in ecosystem function by forming symbiotic networks and facilitating interactions between plants and soil microbes (Sridhar, 2019).

Endophytic fungi are commonly found within healthy plant tissues and represent an essential component of the plant micro-ecosystem. Their presence contributes to improved nutrient absorption, enhanced stress resistance, and overall plant growth (Wen et al., 2022). These fungi exhibit host specificity, as evidenced by the varying species and abundance of endophytic fungi isolated from different plant species. Additionally, these fungi promote root development and improve water absorption, and reduce respiratory losses, helping plants conserve water and surviving arid conditions. A study indicated that endophytic fungi can enhance plant drought tolerance, with colonized plants exhibiting 20%−40% higher survival rates under water stress conditions (Ullah et al., 2019). They also contribute to soil health and ecological functions through interactions with plant root systems (Shah et al., 2024; Wen et al., 2022). Additionally, endophytic fungi can both synthesize bioactive secondary metabolites directly and alter the plant's own metabolic pathways to modulate the production of secondary metabolites, many of which hold industrial significance (Alam et al., 2021; Morales-Vargas et al., 2024). Variations in endophytic fungal communities across different plant species may arise from factors such as plant genetics, environmental conditions, and soil types. In greenhouse and field trials, endophytic inoculation with the *Fusarium culmorum* strain FC-03 (*n* = 6 replicates × 3 sites) resulted in a 15%−30% increase in wheat biomass (mean = 22.4%, SD = 4.7%, *p* = 0.012, two-way ANOVA) under low-phosphorus conditions (Adam et al., 2020). The experiments were conducted over two growing seasons (2018–2019) in loamy soils (pH 6.8), with biomass measured through destructive harvesting at physiological maturity. A recent meta-analysis of 35 peer-reviewed studies (Trentin et al., 2024) reported aweighted mean increase of 28.3% in biomass (95% CI: 22.1%−34.5%, *n* = 1,247 plant samples) and a 37.2% enhancement in stress tolerance (95% CI: 31.5%−42.9%, *n* = 892 samples) under both controlled and field conditions. The analysis utilized a random-effects model (DerSimonian-Laird estimator), with heterogeneity evaluated using I^2^ statistics (*I*^2^ = 68.4% for biomass, *I*^2^ = 52.7% for stress tolerance), indicating moderate variability across studies (Li Y. et al., 2021). Laboratory studies indicatethat endophyte-inoculated maize plants exhibit a 42% reduction in pathogen infection (White et al., 2019). These findings underscore the considerable potential of endophytic fungi in promoting plant growth and resilience.

The bidirectional interaction between plants and endophytic fungi is crucial for ecological adaptation. Plants provide energy to fungi through the photosynthesis of organic matter, while fungi facilitate nutrient absorption and enhance stress resistance (Lugtenberg et al., 2016; Tellez et al., 2022). Endophytic fungi exhibit remarkable adaptability to varying environmental conditions, thriving across various soil types and climatic. This adaptability makes them vital to ecosystems, as they can enhance plant resilience in arid conditions or poor soil, thereby improving plant survival (Aamir et al., 2020). Factors such as soil type, climate, and agricultural practices significantly impact the composition and function of endophytic fungi communities.

### 2.3 Factors influencing endophytic fungal communities

Endophytic fungal diversity and abundance are significantly shaped by abiotic factors. Climate parameters such as temperature, precipitation, and seasonal cycles determine both colonization rate and community composition (Chen et al., 2021). A distinct altitudinal gradient observed in native Hawaiian plants indicates that higher rainfall and moderate temperatures support richer endophytic assemblages (Cobian et al., 2019). Similarly, seasonal fluctuations lead to peak colonization in autumn and the lowest values in winter for cruciferous crops (Chen et al., 2021). Soil physicochemical properties—such as texture, pH, salinity and heavy-metal contamination—further modulate endophyte occurrence. For instance, *Piriformospora indica PI5* maintained stable efficacy in saline soils (EC 8–10 dS m^−1^) and improved maize yield by 35%, demonstrating that well-selected strains can adapt to adverse soil conditions (Case Study 3 in Section 4.4; Li et al., 2019). Furthermore, climate change-driven increases in drought or flooding are predicted to disrupt established communities and iminish their beneficial functions (Martinez-Arias et al., 2023).

Host identity and genotype exert primary control over endophytic communities. Wheat genotypes significantly influence leaf- and root-associated fungi (Lugtenberg et al., 2016), while leaf traits such as thickness and density inversely correlate with endophyte diversity (Tellez et al., 2022). The morphology of root systems is also crucial; for instance, antimicrobial-rich roots of radish suppress colonization rate and diversity (Beevi et al., 2009). Interactions with other microorganisms—whether synergistic or antagonistic—further modify endophytic assemblages (Megan et al., 2010). Across biogeographic scales, endophyte diversity generally follows a latitudinal gradient, peaking at low latitudes, whereas root-associated taxa can exhibit mid-latitude maxima (Barbi et al., 2025).

Agricultural practices significantly influence endophytic communities. The combination of no-tillage and mulching enhances both diversity and abundance within wheat roots (Sharma-Poudyal et al., 2017). In contrast, intensive pesticide and fertilizer applications diminish colonization and species richness, while organic management fosters greater fungal diversity in cruciferous vegetables (Karlsson et al., 2017). Additionally, land-use change and pollution alter host chemistry and stress levels, thereby, indirectly reshaping endophyte composition (Li et al., 2019). Understanding these management effects is crucial for developing endophyte-based strategies that promote sustainable agriculture.

### 2.4 Techniques for the detection and isolation of endophytic fungi

To conduct effective research, it is essential to first isolate and identify these fungi from plant material. Several commonly used methods include: (i) Media techniques: selective media such as malt extraction agar and soil extraction agar can be utilized to isolate endophytic fungi from plant samples. The benefit of this method lies in its ability to produce pure cultured fungal strains for further studies (Poveda et al., 2022; Russo M. et al., 2019). Nevertheless, cultivation recovers only an estimated 1%−15% of the in-planta mycobiome—the “great plate count anomaly” of endophyte ecology—because many symbionts are obligate biotrophs, require unknown signals or enter a viable-but-non-culturable state once outside the host apoplast. Repeated sub-culturing can also select for laboratory-adapted morphotypes that lose ecologically relevant plasmids or cryptic secondary-metabolite clusters, thereby underestimating functional potential (Poveda et al., 2022; Russo M. et al., 2019). (ii) Molecular techniques: high-throughput sequencing and PCR methods are employed to detect the DNA of endophytic fungi. These approaches can reveal the diversity of fungi found in plant samples and provide genomic data that supports taxonomic and functional investigations (Morales-Vargas et al., 2024). Limitations include primer bias (e.g., ITS1F under-represents *Mortierellomycota*), short-read chimeras and DNA from dead or dormant cells that inflate alpha-diversity. Extraction kits also differ in cell-wall lysis efficiency; bead-beating at 6 m s^−1^ for 60 s with 0.1 mm zirconia spheres increases Ascomycota recovery by 20% but shears high-molecular-weight DNA, reducing assembly contiguity. Finally, high-throughput sequencing remains cost-prohibitive for routine monitoring in low-resource settings and demands rigorous bioinformatics pipelines (DADA2, UNOISE3) that are still evolving (Morales-Vargas et al., 2024; Akram et al., 2023). (iii) Staining methods: Specific dyes facilitate the observation of the growth and distribution of endophytic fungi through microscopy. This approach effectively visualizes endophytic fungi within plant tissues (Akram et al., 2023). Drawbacks include laborious sample preparation, potential autofluorescence of phenolic-rich tissues and the requirement for axenic transformants—impossible for unculturable taxa. Moreover, staining intensity correlates with cell-wall thickness, leading to underestimation of early, thin-walled germ tubes. Finally, microscopy is inherently low-throughput; quantifying FHV for 100 root segments requires ~8 h of CLSM time, making it impractical for large ecological surveys unless coupled with automated slide-scanning platforms.

## 3 Effects of endophytic fungi on crop growth

**Figure 2:**
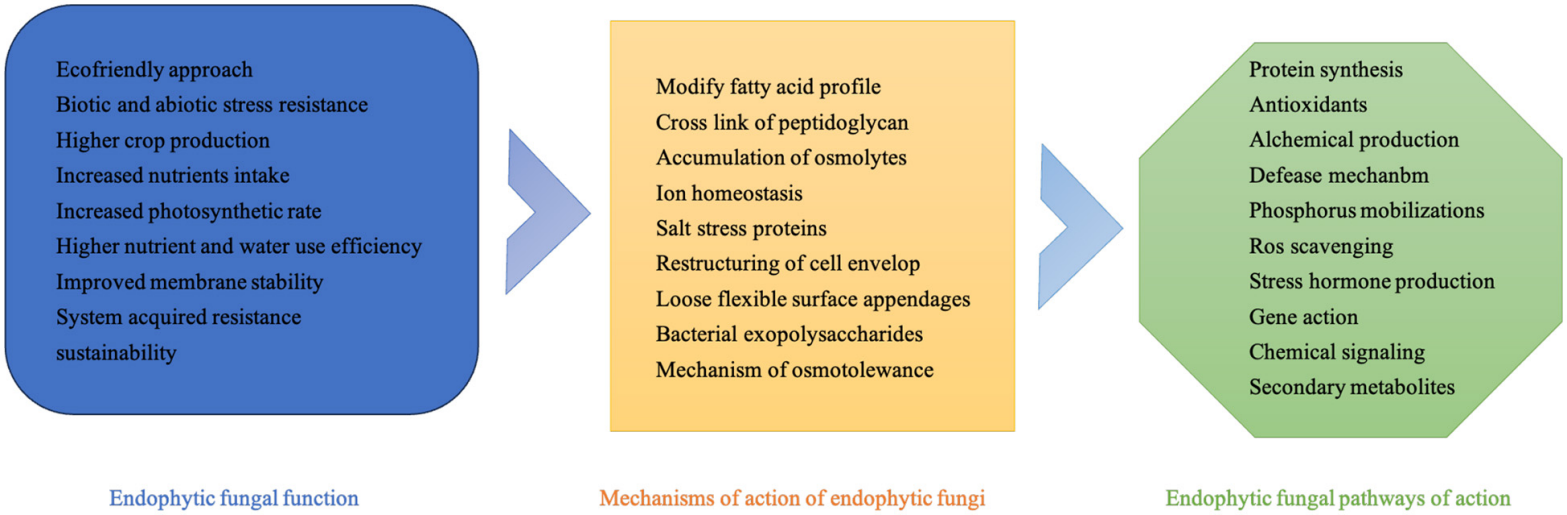
Hierarchical mechanisms of endophytic fungi-mediated stress tolerance and growth promotion in host plants (with drought tolerance as a representative case).

Endophytic fungi, which reside within plant tissues, play a significant role in promoting plant growth and development. Researchers typically employ a variety of inoculation techniques, including foliar sprays, seed immersions, and root applications, to investigate their distinct effects on crop progression (Verma et al., 2024). These fungi contribute to the enhancement of plant growth through multiple mechanisms, encompassing stress tolerance (e.g., drought, salinity) and growth-promoting traits (e.g., nutrient uptake, hormone regulation), as visualized in the hierarchical framework (Figure 2).

### 3.1 Synergistic mechanisms of root development and nutrient acquisition

Endophytic fungi can enhance crop productivity by facilitating root architectural development and nutrient acquisition, but this effect is often constrained by soil nutrient status and host genotype and nutrient acquisition. Their hyphae penetrate soil microaggregates, expanding the root absorptive surface by 20%−50% (Verma et al., 2021), while secreting organic acids such as gluconic acid, to solubilize recalcitrant phosphorus (Yang et al., 2021). These fungi tend to improve soil structure and promote root development in nutrient-poor environments, but may show neutral or negative effects in nutrient-rich soils (Fite et al., 2023; Mahmood and Kataoka, 2020). By expanding the root surface area, endophytic fungi enhance the plant's capacity to access soil nutrients. The hyphae extend into the soil, capturing essential nutrients such as nitrogen, phosphorus, and potassium that plants might otherwise struggle to obtain (Omomowo and Babalola, 2019; Verma et al., 2021). Certain endophytic fungi stimulate systemic root growth, thereby enhancing the absorption of nutrients and water-particularly nitrogen and phosphorus which improves plant competitiveness in resource-scarce environments (Rodriguez et al., 2009). They also assist in mineral absorption from nutrient-deficient soils (Aamir et al., 2020; Bogas et al., 2022). Furthermore, some endophytic fungi fortify plant cell walls, thereby increasing resistance to pathogens (Fontana et al., 2021; Lugtenberg et al., 2016). Specifically, root-associated endophytic fungi, such as *Serendipita indica* and arbuscular mycorrhizal (AM) fungi enhance root architectural development by promoting root hair growth and branching, which increases root surface area and improves water and nutrient uptake (Liu et al., 2023; Tellez et al., 2022). The production of IAA and cytokinins triggers lateral root initiation and root hair elongation (Leetanasaksakul et al., 2024). By increasing IAA levels, these fungi promote root cell expansion and division, driving overall root growth. They also enhance root respiration, optimize oxygen use, and accelerate root growth (Extremadura et al., 2021). This benefits root system and overall plant growth. Furthermore, endophytic fungi boost root microbiota diversity and stability, supporting robust root development (Poveda et al., 2022). Moreover, the interactions between inter-root microorganisms and endophytic fungi can enhance nutrient uptake by plants, ultimately leading to improved growth (Akram et al., 2023; Morales-Vargas et al., 2024). Endophytic fungi directly contribute to crop growth and yield by enhancing nutrient uptake and utilization efficiency (Compant et al., 2016). Enzymatic nutrient mobilization occurs through phytases and phosphatases, which release bound nutrients (Ma et al., 2023; Rodriguez et al., 2009). These enzymes facilitate more effective nutrient absorption by decomposing soil organic matter (Watts et al., 2023). Furthermore, endophytic fungi reduce the viability of pathogens by diminishing antibiotics and bioactive compounds thereby enhancing both plant health and nutrient uptake efficiency (Akram et al., 2023; Sarkar et al., 2021). These fungi are particularly important for nutrient absorption in challenging environments, such as those characterized by low nutrient availability or high salinity. By releasing organic acids and enzymes, they enhance soil nutrient accessibility, promoting robust root system development and overall plant growth (Gimenez Mariño et al., 2007; Trentin et al., 2024). Through their symbiotic relationship with plant roots, endophytic fungi play a vital role in the absorption of essential mineral nutrients by host plants.

Specifically, root-associated endophytic fungi such as *Serendipita indica* and *Arbuscular Mycorrhizal* (AM) fungi promote root hair growth and branching by producing IAA and cytokinins, triggering lateral root initiation and root hair elongation (Leetanasaksakul et al., 2024; Liu et al., 2023). Elevated IAA levels stimulate root cell expansion and division, while enhanced root respiration optimizes oxygen utilization to accelerate growth (Extremadura et al., 2021). These changes expand the root surface area by 20%−50%, enhancing the absorption of nitrogen, phosphorus, potassium, and other essential nutrients (Verma et al., 2021).

While numerous studies report that endophytic fungi (e.g., *Serendipita indica, Arbuscular Mycorrhizal* fungi) enhance root surface area by 20%−50% and improve nutrient uptake (Verma et al., 2021; Liu et al., 2023), significant variability exists across agroecosystems. For instance, under high-phosphorus soil conditions, inoculation with *Fusarium culmorum* strain *FC-03* failed to promote wheat biomass (Adam et al., 2020), contradicting its positive effects in low-phosphorus environments. This context-dependency is further supported by Trentin et al. (2024), whose meta-analysis revealed that the growth-promoting effect of endophytes was 37% weaker in clay soils than in loamy soils, likely due to reduced hyphal penetration in compacted substrates. Conflicting evidence also emerges from studies on host specificity: *Trichoderma harzianum* consistently enhances phosphorus solubilization in maize (Yang et al., 2021) but shows no significant effect in soybean (Russo M. et al., 2019), suggesting species-specific interactions between endophytes and host root exudates.

### 3.2 Enhancement of photosynthetic capacity

Endophytic fungi facilitate plant growth by modulating various aspects of the photosynthetic machinery (Table 2). Firstly, they significantly enhance the biosynthesis of photosynthetic pigments. For instance, inoculation with *Piriformospora indica*, increases leaf chlorophyll a, chlorophyll b and carotenoid contents by 18%−32% under both optimal and drought-stressed conditions (Verma et al., 2022). Second, endophytes enhance PSII photochemical efficiency. Chlorophyll fluorescence measurements (Fv/Fm, ΦPSII, and ETR) from wheat leaves colonized by *Serendipita vermifer* show 10%−15% higher compared to non-colonized controls, indicating improved quantum yield and electron transport rates (Wang et al., 2024). Third, carbon fixation is stimulated through enhanced Rubisco activity and increased expression of the small sub-unit gene “rbcS.” Metabolomic profiling of maize inoculated with *Trichoderma harzianu* reveals a 21% rise in RuBP regeneration rate and a 26% rise in 3-PGA pool size, consistent with accelerated Calvin-cycle turnover (Yang et al., 2021). Fourth, endophytic fungi elevate NPQ capacity, dissipating excess excitation energy under high light and protecting the photosynthetic apparatus from photodamage (Tellez et al., 2022). Lastly, stomatal conductance (g_s_) and intracellular CO_2_ concentration (C_i_) are improved, thereby enhancing mesophyll diffusion and net CO_2_ assimilation (A) by up to 27% in endophyte-treated trifoliate orange seedlings (Liu et al., 2023). Collectively, these multi-level adjustments optimize photosynthetic efficiency, increase carbon capture, and translate into higher biomass accumulation and grain yield under field conditions (Verma et al., 2022; Wang et al., 2024).

**Table 2:** Mechanisms of endophytic fungal production assistance and references.

**Endophytic fungal**	**Functionality**	**Mechanisms**	**References**
*Serendipita indica*	Root growth promotion	Enhanced root hair elongation, branching; IAA synthesis	Liu et al., 2023; Tellez et al., 2022
*Trichoderma spp*.	Nutrient uptake	Increased root biomass via auxin and cytokinin modulation	Hiruma et al., 2018
*Aspergillus flavus*	Nutrient uptake	Solubilized phosphorus; N/P/K absorption	Li Y. et al., 2021
*Penicillium roqueforti*	Nutrient uptake	Enhanced heavy metal (Cd/Zn) uptake under stress	Ikram et al., 2018
*Exophiala sp. LHL08*	Photosynthetic boost	Chlorophyll content; CO_2_ assimilation under drought	Morales-Vargas et al., 2024
*Piriformospora indica*	Hormonal regulation	Gibberellin and cytokinin levels; promoted shoot elongation	Murphy et al., 2014
*Fusarium sp*.	Drought resistance	Proline accumulation; water-use efficiency	Shah et al., 2024
*Chaetomium globosum*	Disease suppression	Produced antifungal compounds (chaetoglobosins); reduced *Fusarium* infection	Farhat et al., 2023

### 3.3 Strengthening host resistance

By examining how these fungi collaborate to promote plant growth, scientists can develop more effective microbial mixtures to enhance crop productivity and stress resistance (Fadiji and Babalola, 2020b). These fungi assist plants in adapting to adverse environmental conditions by fostering the synthesis of resilient compounds (Khiralla et al., 2016; Luis and Cherigo, 2011). Furthermore, endophytic fungi can influence the expression of plant genes, particularly those associated with stress tolerance, nutrient absorption, and growth. This genetic-level regulation enables plants to better adapt to environmental changes, thereby improving their ecological adaptability (Rodriguez et al., 2009; Tellez et al., 2022).

Endophytic fungi play a crucial role in enhancing plant resilience to various environmental challenges, including drought, salinity, and heavy metal contamination. Field evidence confirms this: *Serendipita indica SJ1* enhanced wheat drought tolerance in rain-fed regions (18%−22% yield increase), while *Piriformospora indica PI5* alleviated salinity stress in maize (42% improvement in K^+^/Na^+^ ratio) (Case Studies 1 and 3 in Section 4.4). Certain endophytic fungi can interact with soil heavy metals by producing metal flocculants or chelating agents, protecting plants from potential harm (Morales-Vargas et al., 2024). Additionally, these fungi improve plant resilience to salt stress by enhancing physiological traits and metabolic functions. For instance, some endophytic fungi facilitate the removal of excess salts by improving the root system's ion-selective uptake capabilities and promoting salt transport and excretion (Morales-Vargas et al., 2024). In terms of osmoregulation, endophytic fungi contribute to maintaining cellular water balance under salt stress by promoting the production of osmoregulatory compounds such as proline and sugar alcohols (Kamran et al., 2022; Morales-Vargas et al., 2024). For example, *Exophiala sp. LHL08* from cucumber roots enhances plant growth and stress resilience under salt and drought conditions (Morales-Vargas et al., 2024). Under drought or salt stress, ROS, production increases, causing oxidative damage. Endophytic fungi mitigate this stress by producing enzymes, including SOD, CAT, and APX.

Enhancing these enzymes effectively removes reactive oxygen species and minimizes damage to plant cells, thus increasing plant survival in challenging conditions (Kamran et al., 2022; Morales-Vargas et al., 2024). Endophytic fungi contribute to plant resistance against salt and drought by enhancing antioxidant capacity and improving water retention. For instance, specific endophytic fungi elevate antioxidant enzyme activity in plants, mitigating oxidative stress damage (Ameen et al., 2024). This enhancement increases the resilience of plants, allowing them to continue growing in adverse environments, protecting yield and quality (Su et al., 2021; Watts et al., 2023). Such adaptive response not only enhance growth performance and stress resistance, but also leading to higher crop yields and quality (Lugtenberg et al., 2016). Furthermore, endophytic fungi alleviate salt stress by regulating ionic balance and promoting the accumulation of osmotic substances plant cells (Kamran et al., 2022; Xiong et al., 2012). In addition, these fungi enhance plant tolerance to various environmental stresses, including drought, high salinity, and extreme temperatures (Morales-Vargas et al., 2024). By influencing the plant's metabolic and physiological state, these fungi promote consistent crop growth and increase the capacity to withstand adverse conditions of plant (Tellez et al., 2022). This enhanced resilience enables plants to sustain healthy growth in varying environments, contributing to soil stability and health (Abdul Latif et al., 2013). Research has demonstrated that these fungi enhance plant stress responses and support growth under both biotic and abiotic stresses (Hongyun et al., 2021). Additionally, these fungi are adept at producing plant growth hormones like IAA, which aids root growth and differentiation (Leetanasaksakul et al., 2024).

The stress tolerance enhancement by endophytes is highly context-dependent, with conflicting results reported under different stress intensities. For example, *Exophiala sp. LHL08* significantly improves drought tolerance in cucumber at moderate water deficit (50% field capacity) but loses efficacy under severe drought (20% field capacity) (Morales-Vargas et al., 2024). Similarly, while some studies demonstrate that endophytes reduce pathogen infection by 42%−65% (White et al., 2019), others report increased susceptibility: *Colletotrichum spp*., classified as Class 3 endophytes, can switch from enhancing photosynthesis under low-light conditions to inducing leaf necrosis in tropical trees when host plants are stressed by high temperature (Bamisile et al., 2018). This dual role highlights the need to consider environmental triggers in assessing endophytic functions.

### 3.4 Modulation of plant hormone levels and biosynthesis of bioactive compounds

Endophytic fungi additionally influence plant growth and development by modifying the hormonal equilibrium within plants (Morales-Vargas et al., 2024) (Table 3). They synthesize growth-regulating substances, including plant hormones, cytokinins, and gibberellins, which are critical for plant development (Shah et al., 2024). Under salt and drought stress, robust root systems are essential for optimal water and nutrient absorption, making hormone synthesis crucial for plant growth (Nombamba et al., 2024; Zhang et al., 2023). They also promote cellular activity in plants, increasing growth potential (Rigobelo and Baron, 2022). Hormonal regulation accelerates plant growth and fortifies disease resistance (Rodriguez et al., 2009). Endophytic fungi interact with plants by releasing particular signaling molecules that modulate growth, development, and defense mechanisms. For example, fungal metabolites can trigger plant immune responses, enhancing resistance to pests and diseases (Tellez et al., 2022). They also reduce disease incidence by suppressing pathogenic microbes (Singh and Dubey, 2018). By competing for resources and space, endophytic fungi indirectly mitigate harmful bacterial infections (Burragoni and Jeon, 2021). This interaction is a key area of current research. Endophytic fungi activate plant immune responses against pathogens, improving resistance (Burragoni and Jeon, 2021). Research indicates they significantly reduce diseases such as stem rot in wheat and soybeans by enhancing disease resilience (Waqas et al., 2015a). They also trigger systemic immune responses, inducing systemic acquired resistance (SAR) and increasing overall pathogen resistance. This is achieved through signaling molecules that stimulate the expression of defense-related genes (Malarvizhi et al., 2023).

**Table 3:** Representative endophytic fungi and their modes of action verified at different experimental scales.

**Endophytic fungi**	**Mechanisms**	**References**
*Alternaria*	Regulate phytohormone biosynthesis (IAA production)	Tedersoo et al., 2014
*Ampelomyces sp*.	Promote root development; enhance abiotic stress tolerance (drought/salinity); boost nutrient uptake (N/P/K)	Nouh, 2019
*Bipolaris*	No clear beneficial endophytic mechanisms (only reported as a source of root rot)	Tedersoo et al., 2014
*Cladosporium*	No clear beneficial endophytic mechanisms (only reported as a source of leaf spot disease)	Bensch et al., 2018; http://www.apsnet.org/publications/commonnames/Pages/default.aspx
*Cordyceps*	Enhance nutrient uptake; regulate host immunomodulation and antioxidant capacity	Tedersoo et al., 2014
*Acremonium*	No clear beneficial endophytic mechanisms (only reported as a causative agent of maize diseases)	Tedersoo et al., 2014
*Cyphellophora*	Promote nutrient metabolism (biotransformation of steroidal compounds); enrich γ-linolenic acid via mycelial synthesis	Tedersoo et al., 2014; Yang et al., 2018
*Epicoccum*	No clear beneficial endophytic mechanisms (only reported as a pathogen of tobacco leaf spot)	Tedersoo et al., 2014
*Filobasidium*	Promote plant growth; enhance nutrient uptake; partial beneficial effects (counteracts mold rot)	Wu et al., 2023
*Fusarium*	Regulate phytohormone biosynthesis (gibberellins production); enhance nutrient uptake and solubilization	Costa et al., 2012; Prylutskyi et al., 2017
*Gibellulopsis*	No clear beneficial endophytic mechanisms (non-toxigenic but partially pathogenic to hosts)	Tedersoo et al., 2014
*Leptosphaerulina*	No clear beneficial endophytic mechanisms (only reported as a source of peanut leaf scorch/blight)	Copete-Pertuz et al., 2019
*Mortierella*	Enhance nutrient uptake (mycorrhizal symbiosis with forest trees); promote soil nutrient circulation	Ozimek and Hanaka, 2020
*Paraphoma*	No clear beneficial endophytic mechanisms (only reported as a phytopathogen causing stem rot/leaf necrosis)	Gomzhina et al., 2020
*Parastagonospora*	No clear beneficial endophytic mechanisms (only reported as a common pathogen of wheat/rye)	Richards et al., 2019
*Periconia*	Produce bioactive secondary metabolites (terpenoids, polyketides, etc.); promote nutrient uptake	Azhari and Supratman, 2021; Costa et al., 2012; Prylutskyi et al., 2017
*Peyronellaea*	Regulate phytohormone biosynthesis (IAA production)	Tedersoo et al., 2014
*Phaeosphaeria*	No clear beneficial endophytic mechanisms (only reported as a common pathogen of wheat/rye)	Tedersoo et al., 2014
*Phoma*	Enhance abiotic stress tolerance (activation of SOD, CAT, POD; proline accumulation)	Zhou et al., 2021
*Pyricularia*	Regulate ion homeostasis (sodium/potassium channel expression); enhance abiotic stress tolerance (salinity/drought)	Bettache et al., 2017
*Sarocladium*	Regulate phytohormone biosynthesis (IAA production)	Tedersoo et al., 2014
*Schizothecium*	Regulate phytohormone biosynthesis (IAA production)	Tedersoo et al., 2014; Yasmina and Miller, 2022
*Setophoma*	Promote nutrient cycling (biodegradation of aromatic compounds)	Yang et al., 2017
*Serendipita indica*	Promote root development (root hair growth/branching); enhance nutrient uptake (N/P/K); regulate phytohormone biosynthesis (IAA/cytokinins); boost drought tolerance	Verma et al., 2021; Liu et al., 2023
*Trichoderma harzianum*	Enhance nutrient uptake (phosphorus solubilization); regulate phytohormone biosynthesis (IAA/gibberellins); promote root development; boost biotic/abiotic stress resistance	Yang et al., 2021; Dutta and Banerjee, 2024
*Piriformospora indica*	Enhance salt tolerance (ion homeostasis regulation); promote nutrient uptake; regulate phytohormone biosynthesis; improve photosynthetic capacity	Yun et al., 2018; Verma et al., 2022
*Verticillium*	No clear beneficial endophytic mechanisms (only reported as a pathogen of cotton verticillium wilt)	Tedersoo et al., 2014

Mechanisms are classified based on four core functions of endophytic fungi: enhancing nutrient uptake, regulating phytohormone biosynthesis, promoting root development, and boosting resistance to abiotic stresses. “No clear beneficial endophytic mechanisms” indicates that the strain is only recorded as a pathogen or has no verified beneficial effects in endophytic interactions, included for taxonomic reference only.

Endophytic fungi mitigate biotic stress by producing antibiotics and phenolic compounds, defending plants against natural enemies such as herbivorous insects and pathogenic bacteria (Perez et al., 2020). Some endophytic fungi promote compound production or augment plant immune response (Burragoni and Jeon, 2021; Grabka et al., 2022). They also protect host plants by repelling or poisoning herbivorous insects (Burragoni and Jeon, 2021; Watts et al., 2023). Endophytic fungi can suppress plant pathogens and enhance crop disease resistance through various mechanisms. One primary method is the production of antimicrobial substances and enzymes that inhibit the growth of pathogens, including fungi and bacteria, thereby protecting plants from disease (Ali et al., 2024; Verma et al., 2022). These fungi can also compete with pathogens for growth niches and resources, reducing pathogen survival chances (Ali et al., 2024; Tellez et al., 2022). By curtailing the proliferation of harmful microorganisms, endophytic fungi diminish the risk of plant diseases, promoting healthier plant development and supporting soil biological activity (Shah et al., 2024). Well-nourished plants typically enhanced stronger disease resistance, indicating that endophytic fungi can strengthen the plant's entire immune system (Farhat et al., 2023).

Certain endophytic fungi generate biologically active compounds that stimulate plant development and enhance disease resistance (García-Latorre et al., 2023). These bioactive substances not only provide additional protection for plants but also promote robust growth. For instance, synthesize endophytic fungi produce antibiotics and other defensive substances that help plants fight off diseases (Alam et al., 2021). They communicate with host plants through chemical signals, influencing physiological reactions through signaling mechanisms and promote growth and modulating development (Morales-Vargas et al., 2024). Numerous endophytic fungi produce antioxidants and other bioactive materials that safeguard plants from pathogens while promoting their growth. In particular, endophytic fungi associated with medicinal plants can significantly influence the production of plant metabolites, thereby enhancing the medicinal properties of the host plant (Morales-Vargas et al., 2024). These fungi play a crucial role in modulating plant gene expression, improving the plant's ability to respond to environmental stressors. Studies have shown that they can raise the levels of transcription factors critical for salt tolerance, enabling plants to cope more effectively with salt stress (Kamran et al., 2022; Morales-Vargas et al., 2024). Furthermore, by bolstering plant resistance to various challenges, endophytic fungi increase the resilience of plant to both to biotic and abiotic pressures. This not only promotes plant wellbeing but also contributes to the reducing soil degradation and erosion (Jha et al., 2023; Zhong et al., 2022).

Endophytic fungi not only modulate plant development through hormonal regulation but also orchestrate sophisticated molecular networks that conferinduced resistance (IR) against biotic stresses such as insect herbivory and microbial pathogens. At the transcriptional level, colonization by endophytes (e.g., *Trichoderma atroviride, Fusarium solani*) rapidly up-regulatespathogenesis-related (PR) genes—including “*PR-1*”, “*PR-2*” (β*-1,3-glucanase*), and “*PR-5*” (thaumatin-like protein)—via the salicylic acid (SA)-dependent signaling pathway (Anand et al., 2023). Concomitantly, the JA and ethylene (ET) pathways are primed, as evidenced by the elevated expression of “*PDF1.2*” and “*LOX*” genes, which collectively enhance defense against necrotrophic fungi and chewing insects (Grabka et al., 2022). At the post-translational level, endophytic effectors—such as SSCPs—interact with host pattern-recognition receptors (PRRs) to trigger pattern-triggered immunity (PTI). For instance, the endophytic “*Serendipita indica*” secretes the protein SsPTP1, which suppresses early PAMP-triggered ROS bursts while simultaneously potentiating effector-triggered immunity (*ETI*) through *MAP* kinase cascades (*MAPK3/6*) (Hilário et al., 2022). Endophytes also reprogram host metabolism to synthesize defensive secondary metabolites. Colonization of “*Arabidopsis*” roots by “*Penicillium citrinum*” increases camalexin accumulation via transcriptional activation of “CYP71A13” and “PAD3” genes, resulting in a 42% reduction in “*Pseudomonas syringae*” proliferation (Waqas et al., 2015b). Similarly, maize plants inoculated with “*Beauveria bassiana*” endophytes exhibit elevated benzoxazinoid (Bx) levels (e.g., *DIMBOA*), correlating with decreased leaf-feeding damage by “*Spodoptera frugiperda*” (Russo A. et al., 2019). Below-ground, endophytic fungiprime systemic resistance by mobilizing defense signals through the vascular system. Using split-root assays, Li Y. et al. (2021) demonstrated that “*Fusarium equiseti*” triggers *SAR* in wheat against “*Blumeria graminis*,” marked by increased SA levels in distal leaves and enhanced expression of “*PR-1*” and “*WRKY45*.” This systemic response is further reinforced by mycelial networks that facilitate translocation of fungal-derived microRNAs to aerial tissues, thereby silencing host negative regulators of immunity (Xu L. et al., 2022).

## 4 Effective utilization of endophytic fungi

**Figure 3:**
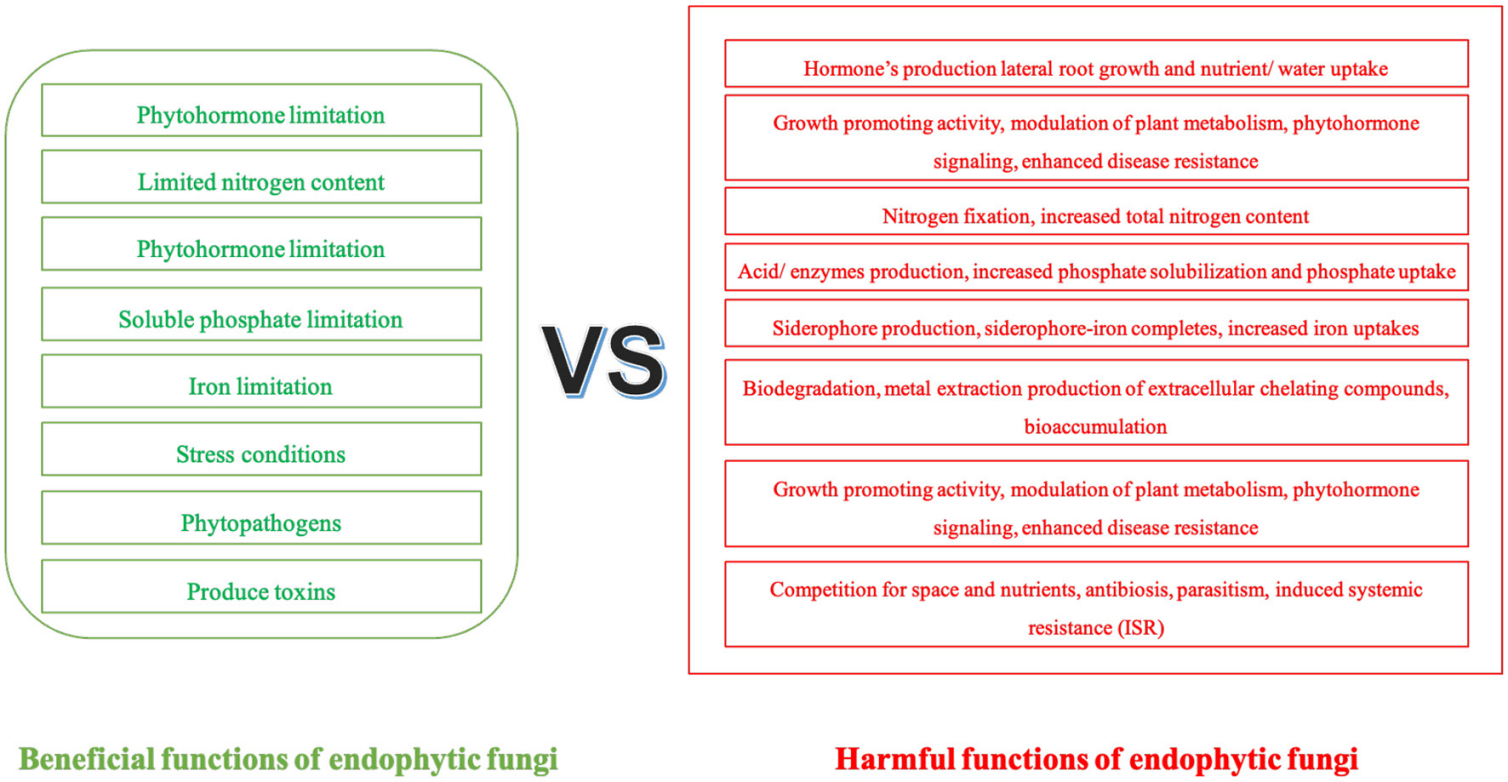
Relative benefits and potential risks of endophytic fungi in agroecosystems.

Endophytic fungi enhance crop growth and boost disease resistance, leading to improved quality and yield. This partnership offers innovative strategies for sustainable agriculture, particularly in addressing challenges such as climate change and soil degradation, with the implementation of endophytic fungi as a promising approach (Tellez et al., 2022). Furthermore, endophytic fungi can reducing reliance on fertilizers and pesticides, thereby lowering production costs (Bamisile et al., 2018; Fadiji and Babalola, 2020a). Their application can decrease the use of chemical fertilizers and pesticides, mitigating the adverse environmental impacts of farming practices. This symbiotic relationship affects ecosystem dynamics by promoting plant development and improving soil health. This association reduces the need for chemical fertilizers but also aids in advancing sustainable agricultural practices (Abdul Latif et al., 2013; Zhong et al., 2022). To effectively enhance agricultural productivity, it is essential to understand the ecological adaptations of endophytic fungi within cross-species symbioses. Endophytic fungi present both benefits and potential risks in agricultural applications. Their beneficial functions include promoting nutrient uptake and enhancing stress resistance, whilst potential risks involve pathogenicity under specific conditions (Figure 3). Their advantageous utilization necessitates rational screening.

### 4.1 Development of stress-resistant crop varieties

By selecting and utilizing specific endophytic fungi, it is possible to develop crop varieties with enhanced resistance to diseases (Lugtenberg et al., 2016; Morales-Vargas et al., 2024; Rodriguez et al., 2009). Researchers can enhance crop resilience and growth by selecting and cultivating varieties that interact favorably with particular endophytic fungi, resulting in higher yields and better environmental adaptability (Aamir et al., 2020; Rigobelo and Cozentino, 2021). For example, *Trichoderma spp*. are well-known for their ability to alleviate abiotic stress and promote plant growth. They enhance plant tolerance to stresses such as drought, salinity, and heavy metal toxicity by producing stress-related enzymes and phytohormones (Hiruma et al., 2018). Similarly, *Piriformospora* has been demonstrated to improve plant growth and yield by enhancing water use efficiency and nutrient uptake (Murphy et al., 2014). These fungi also increase the antioxidant capacity of plants, enabling them to withstand oxidative stress. The application of endophytic fungi (e.g., *Aspergillus flavus*) in crops including soybeans and sunflowers has demonstrated potential for alleviating heat stress (Li Y. et al., 2021). Additionally, other fungi, such as *Rhizopus oryzae*, have been found to increase chlorophyll content, shoot and root lengths, and biomass in host plants subjected to thermal stress.

The strategic utilization of endophytic fungi in agriculture presents significant potential for the development of stress-resistant crop varieties. Such efforts will be essential for achieving sustainable agricultural development and ensuring food security amidst changing environmental conditions.

### 4.2 Advancing sustainable agricultural practices

This research investigates strategies to enhance the utilization of endophytic fungi across diverse agricultural systems and farming environments. These efforts aim to provide scientific support for bolstering sustainable agricultural practices (Abdul Latif et al., 2013; Zhong et al., 2022). Endophytic fungi play a crucial role in nutrient recycling within natural ecosystems. By decomposing organic matter and releasing essential nutrients, they enhance soil fertility, thereby supporting plant development and ecosystem stability (Zheng et al., 2016). The application of endophytic fungi enables farmers to adopt eco-friendly practices, improving crop yield and quality while enhancing soil health and fostering ecological agriculture (Fan et al., 2023). Furthermore, endophytic fungi strengthen plants' defenses against herbivorous pests, reducing the need for reliance on chemical insecticides by providing protective benefits to host plants. This protection may involve the release of substances that attract natural predators or counteract pests (Burragoni and Jeon, 2021). Endophytic fungi serve as biological control agents by suppressing the growth of pathogens and pests. They compete with pathogens for nutrients and space, produce antimicrobial compounds, and activate plant defense mechanisms. This method of biological control reduces reliance on synthetic pesticides and promotes sustainable farming (García-Latorre et al., 2023). Additionally, endophytic fungi decrease the need for chemical fertilizers and pesticides, mitigating their environmental impact and preserving soil ecological balance (Abdul Latif et al., 2013). As bio-fertilizers and biocontrol agents, they reduce chemical inputs, supporting eco-friendly agriculture. Furthermore, they enhance plant nutrient absorption, which lowers farmers' production costs and reduces soil and water contamination from chemical fertilizers (Watts et al., 2023).

Endophytes are diverse fungi that significantly shape plant communities. They improve plant population diversity and stabilize ecosystems by enhancing plant viability. These relationships promote plant health and are vital for maintaining ecosystem function and biodiversity (Rodriguez et al., 2009). Endophytic fungi also collaborate with other microbes such as bacteria to enrich soil biodiversity. This increased diversity strengthens the resilience of soil ecosystems against diseases and environmental stressors (Watts et al., 2023).

### 4.3 Biofertilizers and biocontrol potential

Endophytic fungi are extensively utilized in the field of biological control, both laboratory experiments and field studies have evaluated their effectiveness against these pathogens (Reis et al., 2022). Endophytic fungi inhibit the development of pathogen and pests through mechanisms such as competition and antibiotic production, protecting plants from disease (Akram et al., 2023; Fite et al., 2023). For example, specific endophytic fungi can improve plant disease resistance by directly eliminating pathogens or triggering defensive responses in plants (Zheng et al., 2016). Additionally, by employing strategies like competition, antibiotic production, and parasitism, they combat pathogens and strengthen plant defenses against insects by altering physiological conditions (Rana et al., 2019). This multifaceted protection helps plants remain healthy despite diverse challenges. Additionally, endophytic fungi exhibit bioherbicidal properties, inhibiting weed growth while promoting crop development. This dual capability makes endophytic fungi effective biocontrol agents, decreasing dependence on synthetic herbicides and minimizing environmental pollution (Fite et al., 2023).

### 4.4 Case studies of successful field applications of endophytic fungi

The large-scale field trials summarized below not only demonstrate the practical potential of endophytic fungi in agriculture but also provide concrete evidence to address key challenges such as scalability, environmental adaptability, and technical standardization—critical bottlenecks discussed in previous sections. These cases validate the feasibility of endophytic fungi application under diverse edaphoclimatic conditions and offer insights for overcoming real-world implementation barriers.

Case Study 1: Drought mitigation in winter wheat—Validating environmental adaptability and scalability in rain-fed regions.

A 3-year (2020–2022) drought-mitigation experiment was conducted on 150 ha of winter wheat (*Triticum aestivum* L.) in Hebei Province, a typical rain-fed area of the Huang-Huai-Hai Plain with frequent “winter-spring consecutive droughts.” Seed coating with the Serendipita indica strain SJ1 (1 × 106 CFU g^−1^ seed) resulted in an 18%−22% increase in grain yield under rain-fed conditions, while simultaneously enhancing water-use efficiency by 25% (Li Z. et al., 2021). These improvements were supported by a 30% increase in root length density and enhanced osmotic adjustment through proline and soluble sugar accumulation—physiological responses consistent with the drought tolerance mechanisms analyzed in Section 3.3. The intervention translated into annual irrigation-cost savings of US$120 ha^−1^.

Argument Linkage Analysis: this case directly validates the environmental adaptability of endophytic fungi in region-specific stress scenarios (winter-spring drought in the Huang-Huai-Hai Plain). The 150-ha scale and 3-year duration demonstrate that endophytic fungi can maintain stable efficacy in large-scale field production, addressing the scalability concern raised in Section 5.1. Additionally, the seed coating technique used in this study provides a practical solution for standardized application, responding to the technical standardization demand discussed in Section 4.2.

Case Study 2: Biocontrol of Fusarium wilt in tomato—Overcoming soil-specific pathogen pressure and chemical input dependence.

A two-year (2019–2021) biocontrol trial was implemented on 80 ha of processing tomato (*Solanum lycopersicum* L.) in Andalusia, Spain, where fields were severely afflicted by Fusarium wilt (*Fusarium oxysporum f. sp. lycopersici*). Root dipping of seedlings in Trichoderma harzianum T22 (1 × 107 CFU mL^−1^) resulted in a 65% reduction in disease incidence, outperforming carbendazim-treated plots (50% reduction). Additionally, this treatment increased marketable fruit yield by 28% and Brix by 15% (del Barrio-Duque et al., 2019). Concurrent 16S rRNA profiling revealed a significant enrichment of beneficial taxa (*Bacillus spp., Pseudomonas spp*.) and a concomitant 40% reduction in chemical fungicide input (del Barrio-Duque et al., 2019).

Argument linkage analysis: This case addresses two core challenges: soil-specific adaptability and reduced chemical dependence. The success of *T. harzianum T22* in *Fusarium-infested* soils confirms that endophytic fungi can adapt to pathogen-rich soil environments, complementing the discussion on soil factor influences in Section 2.3. The 40% reduction in chemical fungicides directly supports the sustainable agriculture framework proposed in Section 4.2, proving that endophytic fungi can effectively replace chemical inputs while maintaining or improving crop quality. The 80-ha scale further reinforces the scalability of endophytic fungi as biocontrol agents.

Case Study 3: Salinity resilience in maize—Adapting to abiotic stress gradients and farmer adoption feasibility.

A salinity-resilience trial was conducted from 2021–2023 on 100 ha of maize (*Zea mays* L.) in Punjab, Pakistan, where soils were moderately saline (EC 8–10 dS m^−1^). Seed biopriming with Piriformospora indica strain PI5 (1 × 105 CFU g^−1^ seed) elevated grain yield by 35% despite saline irrigation, improved the K^+^/Na^+^ ratio by 42%, and augmented chlorophyll content (Yun et al., 2018). Post-trial farmer surveys indicated spontaneous adoption by 70% of participants, driven by a 20% reduction in fertilizer expenditure.

Argument linkage analysis: This case provides empirical support for abiotic stress adaptability (salinity) and farmer adoption promotion—two key challenges highlighted in Section 5.1. The ability of *P. indica PI5* to regulate ion balance (K^+^/Na^+^ ratio) aligns with the salt tolerance mechanisms discussed in Section 3.3, validating that endophytic fungi can mitigate specific abiotic stresses in problematic soils. The high farmer adoption rate (70%) addresses the “limited farmer acceptance” bottleneck, as the reduced fertilizer cost directly enhances the economic feasibility of endophytic fungi application—an important consideration for industrial promotion discussed in Section 4.3.

Collectively, these cases underscore three critical insights: (1) Environmental adaptability: endophytic fungi can be tailored to region-specific stresses (drought, salinity, pathogen pressure) through strain selection, addressing the context-dependency challenge in Section 5.1; (2) Scalability: seed-based application techniques (coating, biopriming) enable large-scale deployment (80–150 ha), overcoming the technical scalability barrier; (3) Practical feasibility: economic benefits (cost savings, yield increases) drive farmer adoption, facilitating the industrial promotion discussed in Section 4.3. These empirical findings strengthen the core argument that endophytic fungi can be effectively translated into sustainable agricultural practices by addressing key implementation challenges.

While the three case studies demonstrate the potential of endophytes in drought mitigation, biocontrol, and salinity resilience, their scalability is constrained by context-specific limitations. These results highlight that endophytic applications require site-specific optimization, and generalized recommendations may overestimate their real-world efficacy.

## 5 Conclusion and future prospect

### 5.1 The challenges and limitations of endophytic fungal research

Despite significant progress, endophytic fungal research faces challenges across three core dimensions, with corresponding future research priorities:

(a) Scientific cognition gaps: the molecular mechanisms underlying endophytic fungi-crop interactions (e.g., effector-host receptor recognition) remain incompletely understood. Long-term ecological impacts, especially on soil microbial communities and ecosystem stability, lack systematic evaluation. Future studies should integrate multi-omics approaches (transcriptomics, proteomics, metabolomics) to dissect host defense pathway regulation by endophytes, use CRISPR/Cas9 and split-luciferase assays to clarify receptor-effector interactions, and quantify trade-offs between growth promotion and defense allocation under combined stresses.

(b) Technological application barriers: Inoculation techniques (e.g., dosage, timing, methods) lack standardization, and effectiveness varies with soil types, climates, and crop varieties (Lugtenberg et al., 2016). Future efforts need to optimize application protocols for different agroecosystems and develop specialized biofertilizer products tailored to specific crops and environmental conditions (Ali et al., 2024; Poveda et al., 2022).

(c) Industrial promotion obstacles: Farmers and practitioners have limited understanding of endophytic fungi's mechanisms, hindering their adoption as biofertilizers or biocontrol agents (Rana et al., 2020). Commercialization is also constrained by inconsistent supply chains, inadequate marketing strategies, and underdeveloped regulatory frameworks lacking standardized guidelines and certification processes (Chaudhary et al., 2022; Watts et al., 2023). Addressing these issues requires interdisciplinary collaboration between researchers, industry, and policymakers to improve knowledge dissemination, optimize product development, and establish regulatory systems.

Addressing these issues requires interdisciplinary collaboration between researchers, industry, and policymakers to improve knowledge dissemination, optimize product development, and establish regulatory systems.

### 5.2 Critical synthesis of conflicting evidence and future directions

The application of endophytic fungi in agriculture is hindered by inconsistent results across studies, primarily driven by three factors: (1) Intraspecific fungal variability: Different strains of the same endophytic species (e.g., *Piriformospora indica PI5 vs. PI10*) exhibit divergent effects on crop growth, with *PI5* improving maize yield by 35% under salinity (Yun et al., 2018) while *PI10* shows no significant effect (Murphy et al., 2014). (2) Host genotype specificity: Endophytes that promote growth in modern crop cultivars often fail to do so in traditional landraces, likely due to differences in root exudate composition and immune response pathways (Lugtenberg et al., 2016). (3) Environmental interactivity: The mutualistic role of endophytes can shift to pathogenic under extreme stress (e.g., severe drought, heavy metal contamination), as demonstrated by *Fusarium equiseti* which induces systemic resistance in wheat under mild drought but causes stem rot under severe water deficit (Li Y. et al., 2021).

Future research should prioritize: (1) Standardized testing protocols across multiple agroecosystems to quantify context-dependency; (2) Genomic and transcriptomic analyses to identify fungal effector genes associated with mutualism/pathogenicity switches; (3) Long-term field trials to assess the ecological risks of endophytic inoculation, such as potential displacement of native beneficial microbes or evolution of more virulent strains. Addressing these gaps will enable more robust and evidence-based application of endophytic fungi in sustainable agriculture.

### 5.3 Conclusion

Endophytic fungi play a crucial role in enhancing stress resistance in crops. They bolster the plant immune system and improve disease resistance through various mechanisms, including the activation of resistance pathways, competitive inhibition, and the promotion of overall plant health. Moreover, these fungi significantly contribute to crop development and stress resilience by increasing plants' tolerance to saline and drought conditions. This not only aids in crop development but also fosters sustainable agricultural practices. The utilization of endophytic fungi as biofertilizers holds substantial promise, providing innovative solutions to enhance agricultural yield by promoting plant growth, enhancing disease resistance, and improving soil quality. Looking ahead, there is considerable potential to broaden the application of these microorganisms in agriculture to enhance crop resilience and productivity. Advancing eco-friendly agricultural practices by harnessing the benefits of endophytic fungi will be pivotal for sustainable agricultural development. Through systematic research and careful implementation, endophytic fungi are anticipated to contribute significantly to sustainability and ecological balance in agriculture worldwide. In conclusion, endophytic fungi possess immense potential to advance sustainable agricultural practices, enabling farmers to achieve higher crop yields and improve quality by fostering plant growth, enhancing stress resistance, and supporting soil health. The successful application of endophytic fungi in sustainable agriculture relies on an integrated approach addressing scientific, technical, industrial, and ecological challenges. By prioritizing context-specific mechanism research, standardized application technologies, industrialization, and long-term ecological monitoring, endophytic fungi can be effectively translated into a reliable tool for enhancing crop productivity and resilience under changing environmental conditions.
